# Periodontitis and cardiometabolic disorders: The role of lipopolysaccharide and endotoxemia

**DOI:** 10.1111/prd.12433

**Published:** 2022-03-04

**Authors:** Pirkko J. Pussinen, Elisa Kopra, Milla Pietiäinen, Markku Lehto, Svetislav Zaric, Susanna Paju, Aino Salminen

**Affiliations:** ^1^ Oral and Maxillofacial Diseases University of Helsinki and Helsinki University Hospital Helsinki Finland; ^2^ Folkhälsan Institute of Genetics Folkhälsan Research Center Helsinki Finland; ^3^ Abdominal Center, Nephrology University of Helsinki and Helsinki University Hospital Helsinki Finland; ^4^ Clinical and Molecular Metabolism Faculty of Medicine Research Programs University of Helsinki Helsinki Finland; ^5^ Faculty of Dentistry, Oral & Craniofacial Sciences Kings College London London UK

**Keywords:** microbiota, mouth, oral infections, oral inflammation, plaque, saliva

## Abstract

Lipopolysaccharide is a virulence factor of gram‐negative bacteria with a crucial importance to the bacterial surface integrity. From the host's perspective, lipopolysaccharide plays a role in both local and systemic inflammation, activates both innate and adaptive immunity, and can trigger inflammation either directly (as a microbe‐associated molecular pattern) or indirectly (by inducing the generation of nonmicrobial, danger‐associated molecular patterns). Translocation of lipopolysaccharide into the circulation causes endotoxemia, which is typically measured as the biological activity of lipopolysaccharide to induce coagulation of an aqueous extract of blood cells of the assay. Apparently healthy subjects have a low circulating lipopolysaccharide activity, since it is neutralized and cleared rapidly. However, chronic endotoxemia is involved in the pathogenesis of many inflammation‐driven conditions, especially cardiometabolic disorders. These include atherosclerotic cardiovascular diseases, obesity, liver diseases, diabetes, and metabolic syndrome, where endotoxemia has been recognized as a risk factor. The main source of endotoxemia is thought to be the gut microbiota. However, the oral dysbiosis in periodontitis, which is typically enriched with gram‐negative bacterial species, may also contribute to endotoxemia. As endotoxemia is associated with an increased risk of cardiometabolic disorders, lipopolysaccharide could be considered as a molecular link between periodontal microbiota and cardiometabolic diseases.

## 1 INTRODUCTION

Lipopolysaccharide is an important virulence factor of gram‐negative bacteria. It is often referred to as endotoxin, which is used synonymously with lipopolysaccharide, although there are a few endotoxins that are not lipopolysaccharides.^1^ Virulence is determined as the strength of the pathogenic potential, referring to the relative capacity of a microbe to cause damage in the host and the ability to overcome host defenses.^2^ Several virulence factors or characteristics contribute to the ability of a microbe to cause disease. For example, fimbriae, adhesins, and invasins promote colonization, growth, attachment, and invasiveness. Other factors, such as toxins and proteases, are more immunoinhibitory or immunosuppressive and contribute to tissue‐destructive capacity and evasion of host responses. Also, the susceptibility of the host plays a role in infections.

Lipopolysaccharide resides in the outer membrane of the bacteria, where its hydrophobic structures composed of fatty acid chains anchor the molecule into the bacterial membrane, and the hydrophilic portion (ie the rest of the molecule) projects from the membrane. Lipopolysaccharide is a potent activator of innate and adaptive immune responses, as well as of tissue destruction cascades. It plays a major role in the pathogenesis of periodontitis, where an abundant number of gram‐negative species is a typical determinant of the periodontal microbiota.

Translocation of lipopolysaccharide to the bloodstream causes endotoxemia (ie, lipopolysaccharide activity present in serum/plasma). An approximate twofold increase of lipopolysaccharide activity in apparently healthy subjects is considered ‘metabolic endotoxemia,’ which has been associated with unhealthy nutrition.^3^ Circulating endotoxin is alternatively called ‘intestinal endotoxemia,’ referring to its presumed source, the gastrointestinal tract. The levels reported, for example, among healthy blood donors, middle‐aged subjects, or healthy elderly subjects have been 0.3 pg/ml, 1.2 pg/ml, and 6.7 pg/ml, respectively.^4^, ^5^, ^6^ The corresponding level may reach 850 pg/ml in gram‐negative septic shock caused by lipopolysaccharide.^7^ Human cells expressing the lipopolysaccharide receptor complex are highly sensitive and can respond in minutes to picograms per milliliter of lipopolysaccharide. Chronic endotoxemia is involved in the pathogenesis of many inflammation‐driven conditions, especially cardiometabolic disorders, including atherosclerotic cardiovascular diseases, obesity, liver diseases, diabetes, and metabolic syndrome,e^8^ and thus it is regarded as a risk factor.

Periodontitis patients are known to have increased circulating lipopolysaccharide activity and metabolic disturbances, which may be either the cause or effect of endotoxemia. Observations that bacteria disseminate into circulation after toothbrushing and periodontal procedures^9^, ^10^ and assumptions that endotoxin may disseminate through inflamed periodontium and bleeding gums support the idea of endotoxemia in periodontitis. Bacteremia is not an absolute necessity for the translocation of endotoxins, since lipopolysaccharide may also be secreted as part of the normal membrane vesicle trafficking in bacterial outer membrane vesicles, which contain virulence factors.^11^

This review approaches endotoxemia as a possible molecular mediator between periodontitis and increased risk of cardiometabolic disorders. We describe the structure‐function relationship of lipopolysaccharide, the local and systemic inflammatory and immunological responses caused by lipopolysaccharide, current knowledge on endotoxemia in periodontitis, factors affecting the levels of endotoxemia, and its relation to cardiometabolic disorders.

## 2 STRUCTURE AND FUNCTION OF LIPOPOLYSACCHARIDE AND THEIR RELATIONSHIP

The bacterial cell envelope is essential for the maintenance of cell shape and structural integrity. The cellular wall represents the first line of defense between a bacterium and the host environment. Initial steps that determine the outcome of the interplay between bacteria and the host's immune system greatly depend on the structure and composition of the bacterial cell surface.^12^ Lipopolysaccharide is one of the most studied bacterial surface molecules, renowned for its ability to stimulate the immune system.

**FIGURE 1:**
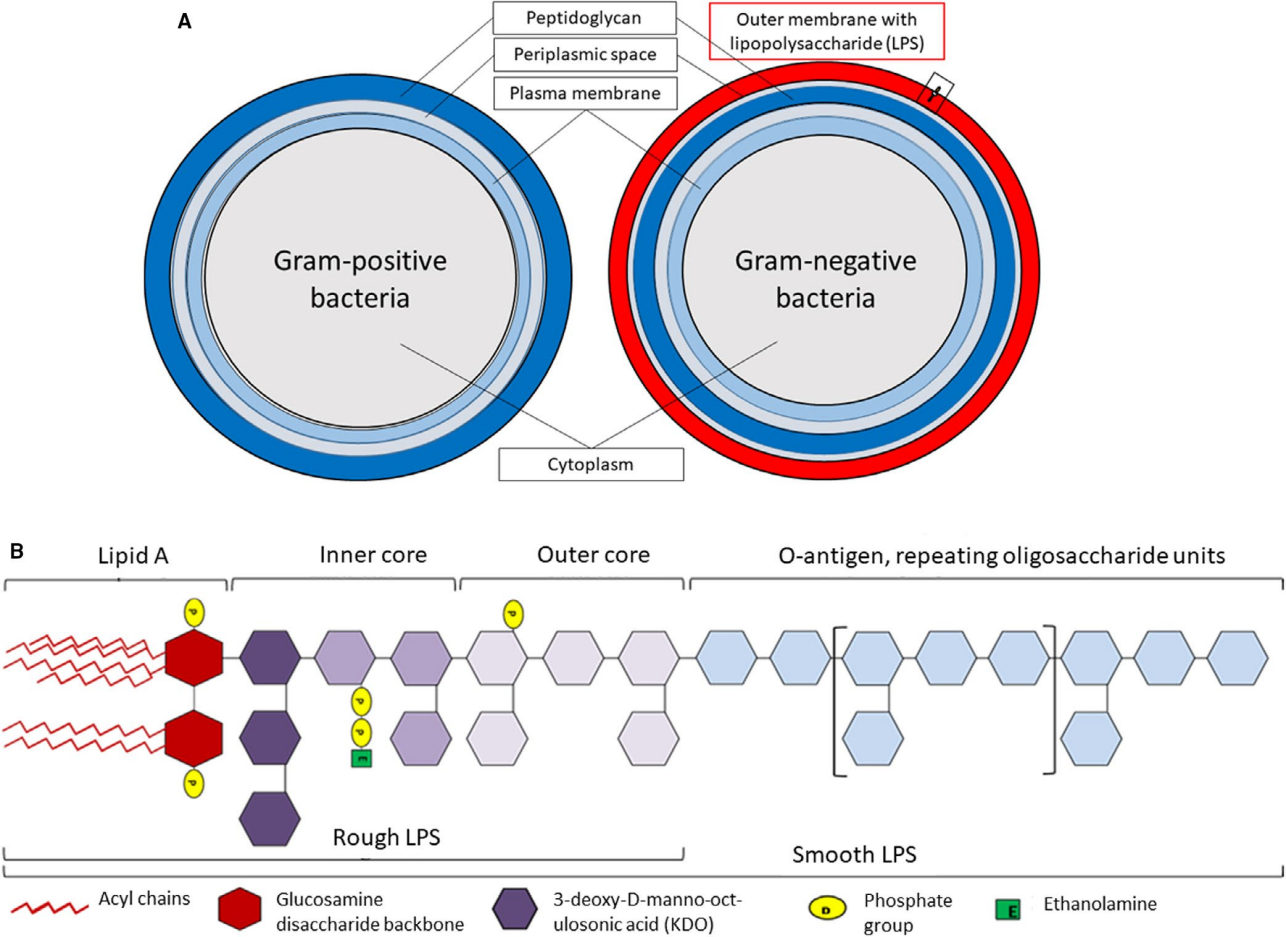
Membrane composition of gram‐positive and gram‐negative bacteria. A, Difference between membrane layers of gram‐positive and gram‐negative bacteria and the presence of lipopolysaccharide (LPS) on the outer membrane of the gram‐negative species. B, General chemical structure of lipopolysaccharide molecule composed of four chemically distinct moieties. Courtesy of Dr Alexander Strachan, University of Plymouth, UK

Depending on whether their cell envelope contains one or two membranes, the prokaryotic organisms can be divided into two main groups: monoderms and diderms.^13^ Most gram‐positive bacteria are bounded by a single cell membrane and a thick peptidoglycan layer. Conversely, gram‐negative bacteria are surrounded by two different cell membranes with a thin peptidoglycan film in the periplasmic space (Figure 1). In contrast to many biological membranes, the outer membrane of gram‐negative bacteria is not a phospholipid bilayer. Instead, it is a highly asymmetric bilayer that contains phospholipids in the inner leaflet and, additionally, lipopolysaccharide molecules in the outer leaflet.^14^ Assembly of these lipids into a continuous barrier, and how that barrier is maintained in response to damage, is a fascinating biological mechanism.

Lipopolysaccharide constitutes 10%‐15% of the total molecules in the outer membrane and represents 75% of the total surface area of gram‐negative bacteria.^15^ The most fundamental role of lipopolysaccharide is to serve as a major structural component of the outer membrane. In addition, lipopolysaccharide molecules transform the outer membrane into an effective permeability barrier against small, hydrophobic molecules that can otherwise cross phospholipid bilayers, making gram‐negative bacteria innately resistant to many antimicrobial compounds.^16^ Lipopolysaccharide can also play a crucial role in bacteria‐host interactions by modulating host immune system responses. It is one of the most conserved structures within all gram‐negative bacterial species. This makes lipopolysaccharide an important pathogen‐associated molecular pattern to be recognized by the highly conserved pattern‐recognition receptors of the mammalian innate immune system, which can subsequently initiate the clearance of a bacterial infection.^17^ In some infections, characterized by the presence of low lipopolysaccharide doses, the immune system responses are beneficial for the host and lead to the rapid clearance of the pathogens. In contrast, in overwhelming infections, higher lipopolysaccharide doses lead to uncontrolled cytokine overproduction and may result in a septic (endotoxic) shock, with unpredictable outcomes.^18^

The function of lipopolysaccharide and its recognition by the host's innate immune system is closely related to its biochemical structure. Lipopolysaccharide consists of three genetically, biologically, and chemically distinct domains (Figure 1):

1. Lipid A, the more or less acylated and phosphorylated glucosamine disaccharide, which is anchored in the bacterial outer membrane.
2. The core oligosaccharide, linked by 3‐deoxy‐d‐*manno*‐oct‐ulosonic acid to lipid A.
3. The so‐called O‐antigen or O‐specific polysaccharide chain, which points to the outside environment.

Lipopolysaccharide that contains all three regions is called smooth (S‐form) lipopolysaccharide, whereas lipopolysaccharide lacking the O‐antigen is named rough (R‐form) lipopolysaccharide or lipo‐oligosaccharides.^19^

Though all three parts of lipopolysaccharide may participate in the modulation of immune activities, lipid A is the primary immunostimulatory moiety of lipopolysaccharide. Endotoxic activity of lipid A depends on the number, length, and saturation degree of acyl chains it contains and on its phosphorylation state.^20^ Lipid A normally consists of a diphosphorylated diglucosamine scaffold bearing several lipid tails. For the full stimulation of the human innate immune system by lipopolysaccharide, it requires the presence of six acyl chains on its lipid A moiety. Lipid A typically contains four primary acyl chains directly attached to the diglucosamine backbone and a variable number of secondary fatty acids linked to the primary ones.^21^ Penta or hepta‐acylated lipid A isoforms are 100‐fold less active, and tetra‐acyl analogues are inactive. Hexa‐acyl lipid A is also necessary for the detection of bacterial invasion by cytoplasmic caspases 4, 5, and 11, which leads to inflammasome activation and consequently to interleukin‐1beta (IL‐1β) and interleukin‐18 secretion, as well as to pyroptosis.^22^ Lipopolysaccharide molecules (eg, from *Escherichia coli*) that trigger a strong proinflammatory reaction of host cells are termed “agonistic” lipopolysaccharides. Structurally different types of lipid A can result in weak inflammatory host responses (“weak agonistic”), or they can even completely block any proinflammatory reactions by competitively binding to corresponding host receptors (“antagonistic lipopolysaccharide”; eg, from *Rhodobacter sphaeroides*).^23^ It has been suggested that the shape of lipid A, described as conical or cylindrical, determines the interaction of lipopolysaccharide with the toll‐like receptors.^24^ A famous example of this is *Porphyromonas gingivalis*, whose less conical lipopolysaccharide may activate different toll‐like receptor signaling pathways.^25^ The remarkable heterogeneity of *P. gingivalis* lipopolysaccharide may enable multiple strategies that the bacterium has to avoid the host defense and contribute to tissue destruction in periodontal pathogenesis.^26^ However, more research is necessary of the role of lipopolysaccharide structure‐function relationship in the periodontal biofilm.

Several bacterial enzymes are involved in the modification of the number of acyl chains or the phosphate groups of lipid A, and a majority of the genes encoding these enzymes are regulated by two global regulatory systems, PhoP/PhoQ and PmrA/PmrB.^27^ The pattern of lipid A phosphorylation significantly affects its endotoxicity and resistance to antimicrobial peptides. Lipid A lacking one or both phosphate groups in *Helicobacter pylori*, *Leptospira interrogans*, *P. gingivalis*, or *Francisella novicida* is less inflammatory and has reduced affinity for antimicrobial peptides compared to with bisphosphorylated lipid A precursors.^28^ Adding a negatively charged phosphate group modifies the lipid A charge and decreases its affinity to the positively charged polymyxins for lipopolysaccharide destabilization. Therefore, altering the bacterial surface charge by adding positively charged moieties to lipid A reduces the binding affinity of cationic antimicrobial peptides to the bacterial outer membrane.^29^ Lipopolysaccharide is also effectively detoxified by human alkaline phosphatases, which remove phosphates from lipid A by dephosphorylation.^30^

The core oligosaccharide region of lipopolysaccharide, containing approximately 10 monosaccharide units, is attached to lipid A and can be further subdivided into an inner core and an outer core. The inner core usually contains l(d)‐*glycero*‐d‐(l)‐*manno*‐heptose and 3‐deoxy‐d‐*manno*‐oct‐ulosonic acid residues, whereas the outer core is usually composed of hexoses. Attached to the outer core are the repeating units of O‐antigen (O‐polysaccharide), which vary in composition, stereochemistry, and the sequence of O‐glycosidic linkages between bacterial strains and thereby give rise to O‐serotype specificity within bacterial species.^31^

At least 20 different sugar molecules may compose the O‐antigen, including molecules that are rarely found in nature, such as abequose, colitose, paratose, and tyvelose. The O‐antigen is the immunodominant part of lipopolysaccharide and, therefore, is the easiest target for the host's humoral response. For this reason, the O‐antigen is the basis for the serological classification of gram‐negative bacteria.^21^ The modifications and the variability of the O‐antigen play an important role in infections, given that they benefit bacteria by influencing adherence, colonization, and the ability to evade host's defense mechanisms.

## 3 QUANTITATION OF LIPOPOLYSACCHARIDE

Lipopolysaccharide could be useful for serological discrimination, as it is a pathogen‐specific biomarker of gram‐negative bacteria. The amount of sample material would be abundant, since a single *E. coli* cell possesses approximately 2 million lipopolysaccharide molecules on its surface.^32^ However, most of the lipopolysaccharide assays developed so far are not able to differentiate between species or serotypes.^33^

The gold standard method for measuring lipopolysaccharide activity is the *Limulus* amebocyte lysate assay, which measures the biological activity of the lipid A portion of the lipopolysaccharide molecule. It is based on the property of an aqueous extract of blood cells (amebocytes) collected from the Atlantic horseshoe crab, *Limulus polyphemus*, to agglutinate through protease cascade reactions upon the addition of endotoxin. The *Limulus* amebocyte lysate reagent can be conveniently linked to a chromogenic substrate in order to enable quantitative measurement. On the one hand, the *Limulus* amebocyte lysate assay is highly sensitive, but on the other hand, it is easily affected by contaminations and inhibitors. As described widely in the present review, circulating lipopolysaccharide activity is neutralized by several plasma proteins. If the sample is not pretreated (eg, dilution, heating, proteases), the *Limulus* amebocyte lysate assay detects the remaining lipopolysaccharide activity, which may actually be clinically more relevant than the total lipopolysaccharide mass.

Technologies based on the binding of lipopolysaccharide to its natural carriers, such as lipopolysaccharide‐binding protein, high‐density lipoprotein, or low‐density lipoprotein, have also been utilized in lipopolysaccharide quantification. After pulling down lipopolysaccharide from the sample, it can be quantified based on its activity by the *Limulus* amebocyte lysate assay or immunoreactivity by specific antibodies. In an “immunolimulus” assay, lipopolysaccharide is first bound to a microtiter well with a capture protein or antibody and subsequently quantified by the *Limulus* amebocyte lysate assay. Other immunoassays used to quantitate lipopolysaccharide are the enzyme‐linked immunosorbent assay, which can be used either to detect lipopolysaccharide antigen or anti–lipopolysaccharide antibodies. The prerequisites for these methods are a careful choice of antigen and production of suitable antibodies, which both need to be characterized.

Biological assays to measure lipopolysaccharide include the “endotoxin activity assay,” which measures the ability of lipopolysaccharide‐antibody complexes to induce the production of reactive oxygen species by blood neutrophils of the patient,^34^ measurement of toll‐like receptor 4 agonists by using transfected human embryonic kidney cells,^35^ or the use of human THP‐1 monocytes with stably transfected nuclear factor‐κB reporter constructs.^36^ In addition, indirect assays to evaluate lipopolysaccharide levels can be used. They include measuring lipopolysaccharide–transferring proteins, such as lipopolysaccharide‐binding protein and soluble cluster of differentiation 14, or the capacity of plasma to neutralize the activity of added lipopolysaccharide.^33^, ^37^ Lipopolysaccharide mass assays include quantitation of 3‐hydroxymyristate (the most abundant hydroxylated fatty acid of the lipid A moiety of most lipopolysaccharide molecules) by combining gas or liquid chromatography with mass spectrometry.^38^, ^39^ The lipopolysaccharide mass has a moderate correlation with the lipopolysaccharide activity determined by *Limulus* amebocyte lysate assay.^39^

## 4 HUMAN MICROBIOME, BACTERIA, AND LIPOPOLYSACCHARIDE

The human microbiome/microbiota consists of bacteria, archaea, eukaryotes, and viruses. Commensal microorganisms colonize all skin and mucosal surfaces. The key role of the microbiota is to protect and maintain the health of the host. Diversity of the species in the microbial community composition reduces the possibilities of colonization for pathogens and is important for the protective function against the shift towards microbial imbalance or dysbiosis.

Commensal microbiota is site specific: Distinct microbes reside in the oral cavity, nasopharynx and respiratory tract, in the gastrointestinal, genital and urinary tracts, in the conjunctiva, and on skin surface. The majority of microbes are located in the gastrointestinal tract. The human digestive system is host to approximately 100 trillion commensal organisms, which makes the gut microbiota the major source of lipopolysaccharide, contributing up to 1 g of lipopolysaccharide as an enteric reservoir.^40^, ^41^, ^42^ The commensal gut flora benefit the host by protecting against pathogens and epithelial cell injury, regulating epithelial development and host fat storage, stimulating intestinal angiogenesis, and instructing innate immunity.^43^ The gut microbiota protects the gastrointestinal mucosa permeability, and in healthy individuals the intestinal barrier function prevents transmission of lipopolysaccharide from gut microbes into the blood.^44^, ^45^, ^46^ The specific commensal flora aims to keep the balance, but changes in the microbiota may lead to dysbiosis and microbiota‐associated diseases. However, it remains to be studied further whether dysbiosis can be effectively treated, if the treatment decreases gut permeability, and whether this lowers endotoxemia and systemic inflammation.

In addition to the gut, the oral cavity presents one of the most diverse microbial communities among the human sites.^45^, ^47^ Indeed, up to 700 bacterial species comprising typically 3000‐7000 different phylotypes (operational taxonomic units) have been identified. The oral microbiome and host factors comprise a feed‐forward loop with appearance and persistence of dysbiosis. This vicious cycle leads to periodontitis and eventually tooth loss. In periodontitis, the controlled immuno‐inflammatory state is broken down and gamma diversity increases, while synergy converts into dysbiosis.^48^ Inflammation is crucial for the development of dysbiosis: It produces essential nutrients for dysbiotic communities, which can subvert specific antimicrobial pathways to enhance their survival.^48^, ^49^ Subgingival plaque is the niche in the oral cavity with the highest richness and diversity of species. The phyla comprising 99% of the total counts are typically gram‐negative Bacteroidetes, Fusobacteria, Proteobacteria, TM7 (Saccharibacteria), and Spirochaetes, and gram‐positive Firmicutes and Actinobacteria.^50^ Dysbiosis is characterized by a relative increase of gram‐negative species in the subgingival plaque.

## 5 INFLAMMATORY AND IMMUNOLOGICAL RESPONSES TO LIPOPOLYSACCHARIDE

### 5.1 Signaling and inflammatory response

The chemotactic gradients created by the bacteria and their virulence factors (including lipopolysaccharide) in the periodontal sulcus lead to inflammatory responses. It has been shown that periodontitis‐associated bacteria (eg, *Aggregatibacter actinomycetemcomitans* and *P. gingivalis*) can invade the host epithelium and underlying connective tissue; and within epithelium, they are able to replicate and spread to neighboring cells.^51^ A tremendous amount of research during the last decades has illustrated the mechanisms by which lipopolysaccharide provokes a strong innate immune response in the human body, both locally and systematically.

**FIGURE 2:**
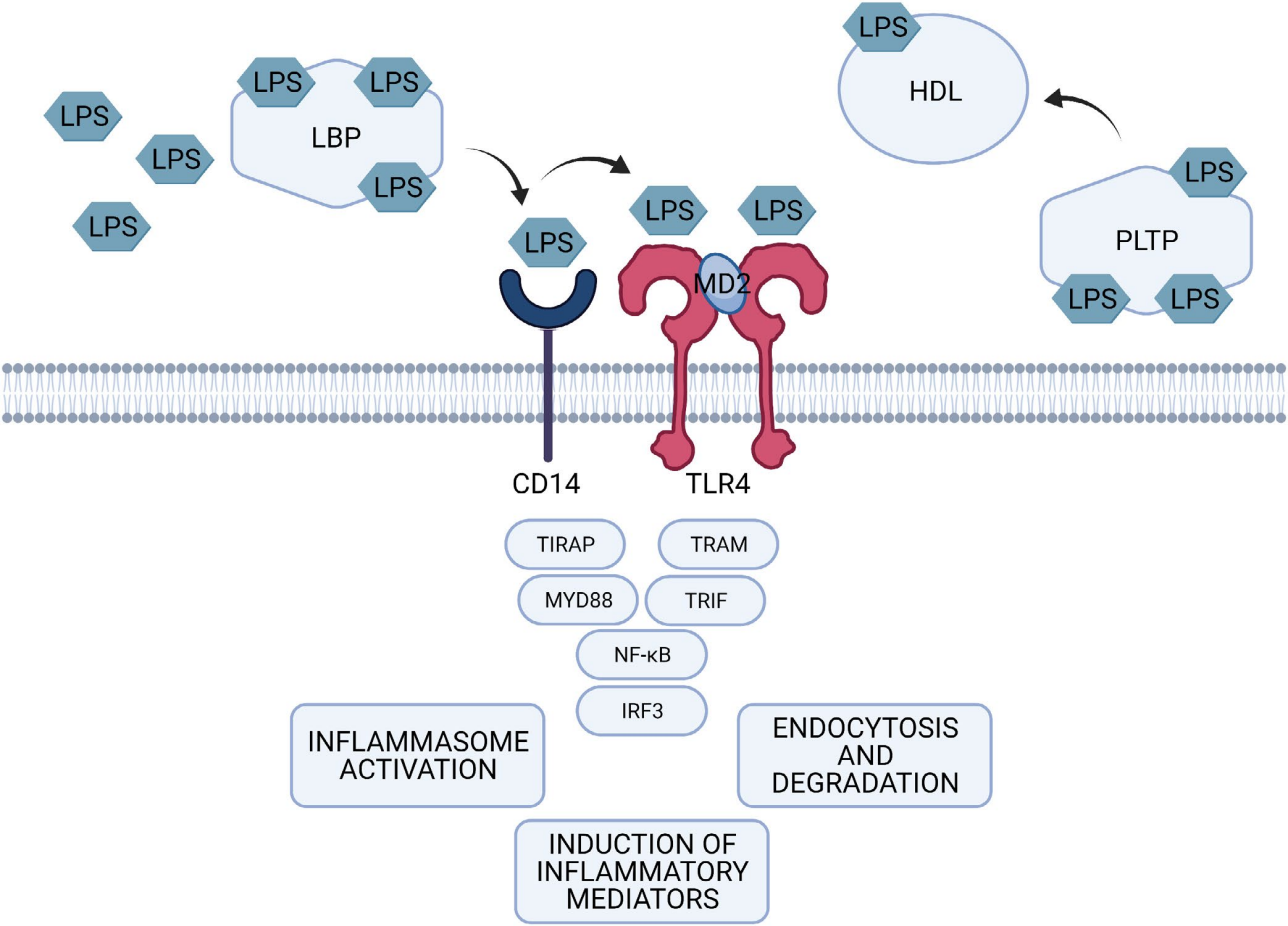
Lipopolysaccharide (LPS)‐toll‐like receptor 4 (TLR4) signaling. Lipopolysaccharide is recognized by the complex of toll‐like receptor 4, cluster of differentiation 14 (CD14), and myeloid differentiation 2 (MD‐2). Toll‐like receptor 4 activation leads to the recruitment of additional effector proteins, including myeloid differentiation primary response protein 88 (MYD88), toll/interleukin‐1 receptor domain‐containing adapter protein (TIRAP), toll/interleukin‐1 domain‐containing adaptor protein inducing interferon‐beta‐related adaptor molecule (TRAM), and toll/interleukin‐1 domain‐containing adaptor protein inducing interferon‐beta (TRIF). These further trigger a cascade enabling nuclear factor‐κB (NF‐κB) and interferon regulatory factor 3 (IRF3) to diffuse into the nucleus and to activate the transcription of cytokines, especially tumor necrosis factor alpha, interleukin‐1beta, interleukin‐6, and interleukin‐8, and interferons, aiming at eliminating pathogens. In the circulation, lipopolysaccharide is transported by lipopolysaccharide binding protein (LBP), phospholipid transfer protein (PLTP), and by lipoproteins. Under standard physiologic conditions, lipopolysaccharide preferentially associates with high‐density lipoprotein (HDL), which contributes to its clearance via the liver and bile

**FIGURE 3:**
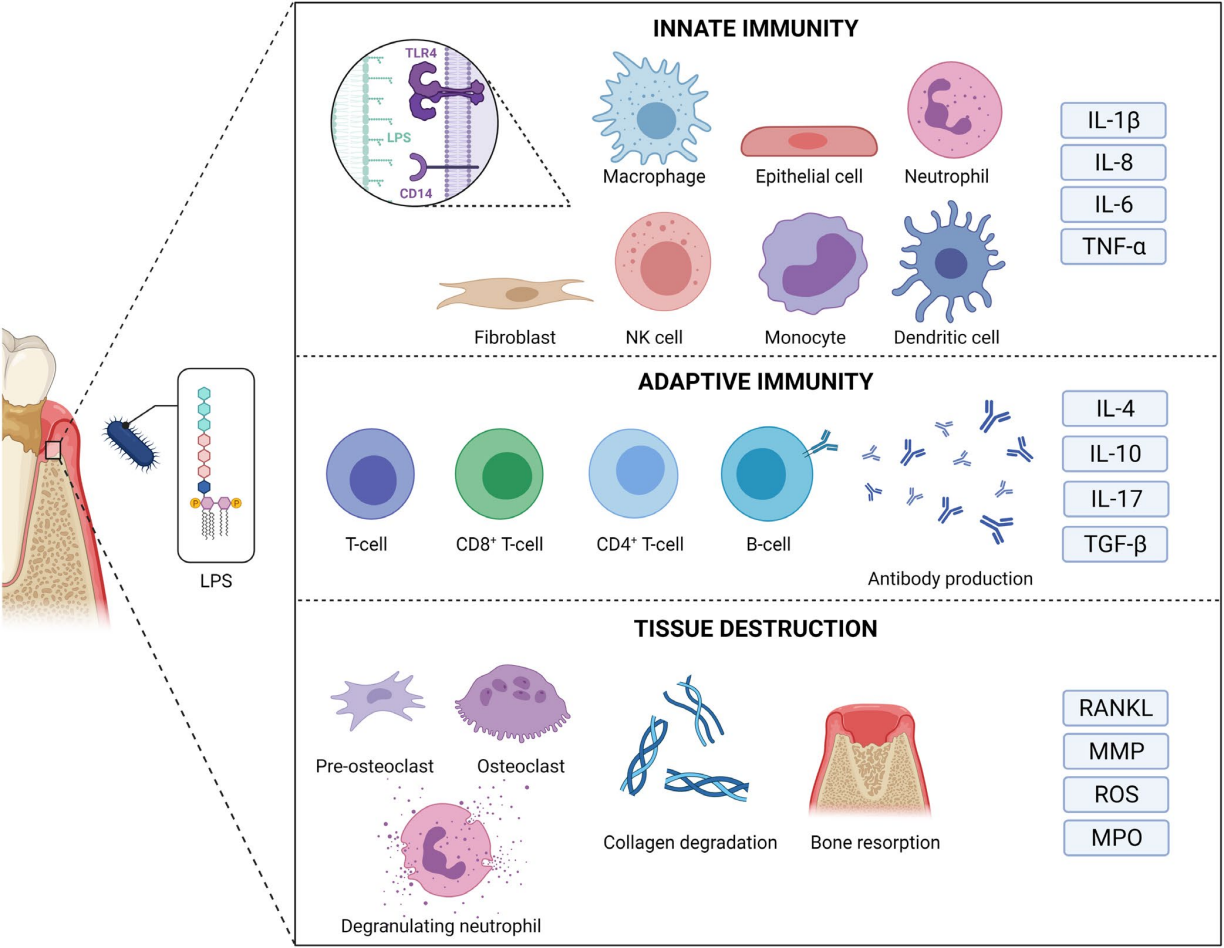
Lipopolysaccharide (LPS) of periodontal bacteria activates the innate and adaptive immune cascades and leads to the destruction of soft and hard tissues of the periodontium, leading to clinical signs of periodontitis. IL, interleukin; MMP, matrix metalloproteinase; MPO, myeloperoxidase; NK, natural killer; TGF, transforming growth factor; TNF, tumor necrosis factor; RANKL, receptor activator of nuclear factor‐κB ligand; ROS, reactive oxygen species

Lipopolysaccharide represents a pathogen‐associated molecular pattern that typically can readily stimulate the innate immune system, which mediates the first‐line local inflammatory response.^52^ Microbes are detected by pattern‐recognition receptors of the host, including C‐type lectin receptors and toll‐like receptors. The bioactive lipid A of lipopolysaccharide, in the form of a single molecule or aggregates, is recognized by the widely studied lipopolysaccharide receptor complex, which comprises toll‐like receptor 4, cluster of differentiation 14, and myeloid differentiation 2 (Figure 2). The complex is expressed on different cell types, including monocytes, macrophages, neutrophils, epithelial cells, and fibroblasts (Figure 3). In addition to membrane‐bound toll‐like receptors, their soluble forms (soluble toll‐like receptors) are present in saliva.^53^ Patients with chronic periodontitis display altered expression profiles of toll‐like receptors and cluster of differentiation 14,^54^ which may indicate dysregulation of host responses associated with periodontitis.

Lipopolysaccharides from oral commensal bacteria are considered mild toll‐like receptor 4 agonists, whereas subgingival biofilm from diseased sites strongly activates toll‐like receptor 4.^55^ *P. gingivalis* synthesizes a heterogeneous population of lipid A structures, which may act in an agonistic, inert, or antagonistic manner with toll‐like receptor 4.^56^ In addition, *P. gingivalis* lipopolysaccharide may also activate toll‐like receptor 2, which typically recognizes pathogen‐associated molecular patterns, such as lipoproteins, lipoteichoic acid, yeast cell‐wall polysaccharides, and peptidoglycan. In fact, this toll‐like receptor 2 activation attributed to lipopolysaccharide or lipid A may be due to other cell‐membrane components copurifying with *P. gingivalis* lipopolysaccharide preparations.^57^, ^58^

Recognition of lipopolysaccharide by lipopolysaccharide‐binding protein initiates the clearance of gram‐negative bacteria from infected host tissues (Figure 2). Lipopolysaccharide‐binding protein is mainly synthetized by hepatocytes, but it can be also produced by nonhepatocytes, such as gingival tissue cells.^59^ A structurally and functionally related salivary protein, parotid secretory protein that is expressed in the parotid glands, submandibular glands, and gingival epithelial cells, may mediate a similar task in the oral cavity.^60^ lipopolysaccharide‐binding protein transports the lipopolysaccharide molecule to membrane‐bound cluster of differentiation 14, which is expressed mainly by macrophages and dendritic cells and to a lesser extent by neutrophils. Liver, enterocytes, and monocytes secrete the soluble form of the receptor cluster of differentiation 14^61^ to mediate lipopolysaccharide‐responsiveness to cells not expressing cluster of differentiation 14. Cluster of differentiation 14 is lacking a transmembrane domain and, therefore, toll‐like receptors are responsible for the downstream signaling pathway inducing cytokine expression through the activity of nuclear factor‐κB transcription factors.^62^ Toll‐like receptor 4 activation leads to the recruitment of additional effector proteins, including myeloid differentiation primary response protein 88^63^ and toll/interleukin‐1 domain‐containing adaptor protein inducing interferon‐beta.^64^ This further triggers a cascade enabling nuclear factor‐κB to diffuse into the nucleus and to activate the transcription of cytokines, especially tumor necrosis factor alpha, IL‐1β, interleukin‐6, and interleukin‐8 aiming at eliminating the pathogen.^56^

In addition, other membrane‐bound and cytosolic receptors are stimulated by lipopolysaccharide. A review by Kieser and Kagan introduced a more complex lipopolysaccharide recognition pattern by innate immunity independently of toll‐like receptor 4.^65^ Interestingly, cluster of differentiation 14 is also required for pathogen‐induced endocytosis of toll‐like receptor 4, thereby harboring a dual role in transporting pattern‐recognition receptors (such as toll‐like receptor 4) and pathogen‐associated molecular patterns (such as lipopolysaccharide).^66^ Recognition of lipopolysaccharide by gingival epithelial cells requires molecular internalization into the cells, and the activation of toll‐like receptor 4 occurs in the endosome.^67^ In addition, these toll‐like receptor 4–independent routes include lipopolysaccharide‐induced assembly of inflammasomes by caspase 11, activation of reactive oxygen species synthesis, and phagocytosis by brain‐specific angiogenesis inhibitor 1.^65^

In addition to lipopolysaccharide, which is the major pathogen‐associated molecular pattern and a natural toll‐like receptor 4 ligand, toll‐like receptor 4 is activated by injury or inflammation‐induced endogenous danger‐associated molecular patterns. Such nonmicrobial agonists of toll‐like receptor 4 are oxidized low‐density lipoprotein and oxidized phospholipids,^68^ which can be formed by inflammatory response and oxidative stress induced by lipopolysaccharide.^69^ Therefore, lipopolysaccharide is involved both in microbial and nonmicrobial patterns to trigger inflammation.

### 5.2 Immunological responses

Lipopolysaccharide acts as an activator and modulator of inflammation, which is characterized by neutrophil infiltration.^70^ Lipopolysaccharide triggers the redistribution of major histocompatibility complex class I and II molecules to the surface of antigen‐presenting dendritic cells.^71^ At the site of infection, the adaptive immune response is controlled by T cells, which regulate B cell differentiation into antibody‐producing plasma cells. Antibodies complement the immune defense against endotoxin (Figure 3). Even low doses of lipopolysaccharide lead to secretion of antibodies, especially immunoglobulin G (IgG) and immunoglobulin M, and lipopolysaccharide is often present in adjuvants to enhance their activity.

The first line of adaptive immune defense of the mucous epithelium in the oral cavity is secretory immunoglobulin A (IgA), which may play an important role in the oral microbiota homeostasis.^72^ Secretory IgA antibodies reduce the adherence of bacteria to the oral surfaces, including mucosa and teeth‐shielding mucosal surfaces, from invasion of pathogens. Secretory IgA is composed of two IgA molecules, a joining protein and a secretory component, whereas plasma IgA is a monomer. Multiple types of cellular IgA receptors have been characterized, and the binding of IgA or IgA immune complexes to their receptors may either dampen excessive immune responses or initiate proinflammatory cellular processes.^73^

The O‐antigen of lipopolysaccharide determines the antigenicity of the bacterial cell and its immunogenic properties leading to the production of antibodies. The broadly conserved lipid A–core‐oligosaccharide moiety is less immunogenic. The typical polysaccharide side chains restrict access of molecules to the bacterial surface.^74^ Thus, millions of lipopolysaccharide molecules on each cell form a barrier that limits contact of antibodies to the bacterial surface. Thus, lipopolysaccharide may escape the host's immune response or result in immunological mimicry promoting harmful auto‐antigenicity.^75^

As the lipid domain of lipopolysaccharide is hydrophobic, the molecule cannot float freely in the circulation. The lipoprotein unbound portion of lipopolysaccharide activity in the circulation may be recovered in vesicles, especially outer membrane vesicles produced by gram‐negative bacteria. In addition to lipopolysaccharide, outer membrane vesicles consist of outer membrane phospholipids and proteins, such as *P. gingivalis* gingipains,^11^ and they are potent immunogens. They do not just bud out from the outer membrane, but their biogenesis is a regulated process.^76^ The outer membrane vesicle phospholipid composition is not the same as the parent outer membrane,^77^ and their lipopolysaccharide profile may also be distinct compared to the parent outer membrane.^78^ Substantial amount of outer membrane vesicles can be retained on the bacterial cell surface, but many outer membrane vesicles are released into the environment. Therefore, outer membrane vesicles have been proposed to act as diffusible vehicles that can distribute multiple molecular effectors, including those involved in host immune modulation and dysregulation, host‐cell interaction, and biofilm formation.^78^ For example, *P. gingivalis* outer membrane vesicles can disturb the host immune responses through the outer membrane vesicle–associated gingipains, which effectively degrade IgG, immunoglobulin M, and complement factor C3,^79^ and cluster of differentiation 14.^80^

### 5.3 Tissue destruction

Lipopolysaccharide contributes to the destruction of periodontal tissues by increasing the expression and release of proteolytic enzymes, such as matrix metalloproteinases, by various cell types, and by stimulating the migration, differentiation, and activation of osteoclasts via toll‐like receptor signaling (Figure 3).^81^, ^82^ Lipopolysaccharide per se may increase matrix metalloproteinase‐1 and matrix metalloproteinase‐9 expression in epithelial cells and macrophages^83^, ^84^, ^85^ and matrix metalloproteinase‐3 and matrix metalloproteinase‐13 expression in periodontal ligament fibroblasts.^86^, ^87^ In addition, the cytokines, chemokines, and prostaglandins induced by lipopolysaccharide stimulate the degranulation of neutrophils, which release, for example, matrix metalloproteinase‐8 and myeloperoxidase to the extracellular space. *P. gingivalis* lipopolysaccharide enhances osteoclastogenesis and bone resorption by stimulating receptor activator of nuclear factor‐κB ligand (RANKL) in osteoblasts via toll‐like receptor 2.^81^ Lipopolysaccharide also upregulates the expression of CXC motif chemokine receptor 4 in pre‐osteoclasts via toll‐like receptor 4, which subsequently stimulates pre‐osteoclast migration. In addition, the upregulation of CXC motif chemokine receptor 4 promotes RANKL‐induced osteoclast differentiation.^82^

At the site of infection and lipopolysaccharide challenge, T helper cell response is strongly stimulated (Figure 3). T helper 17 cells express interleukin‐17, which can potentially induce the expression of matrix metalloproteinases in fibroblasts, endothelial cells, and epithelial cells, as well as RANKL expression in osteoblasts.^88^, ^89^ Thus, interleukin‐17 also mediates the destruction of both soft and hard tissues in periodontitis. Therefore, T helper 17 cell inhibition has been proposed as a promising therapeutic approach in periodontitis.^90^

### 5.4 Endotoxin tolerance

The subgingival microbiota and their virulence factors, both in health and disease, represent a constant challenge to the host immune system, and the maintenance of periodontal health depends on the efficacy of fine‐tuning mechanisms responsible for control of inflammation. The long‐standing belief that immunological memory is the exclusive characteristic of the adaptive immune system has recently been challenged by emerging indications that innate immunity can also maintain memory of past events.^91^ Such immunological modifications can take two opposing forms: trained immunity and tolerance. Trained immunity involves metabolic and epigenetic adaptations of innate immune cells and their hematopoietic progenitors after exposure to certain microbial stimuli, so that the trained cells respond much faster and stronger to a subsequent challenge. In contrast, induction of immune tolerance leads to attenuated immune responses to repeated stimuli.^92^ Given the constant challenge of the local innate immune system by subgingival microbial communities, impaired induction of immune tolerance or pronounced trained immunity could lead to uncontrolled inflammatory responses in periodontal tissues.

Endotoxin tolerance is a refractory state, observed in innate immune cells and other cytokine‐producing cells, aimed at prevention of excessive and prolonged inflammatory response.^93^ When exposed to a repeated challenge with lipopolysaccharide, these cells shift into a state of hyporesponsiveness, resulting in reduced production of selected cytokines. In this regard, endotoxin tolerance provides protection against tissue destruction under constant exposure to microbial virulence factors. Although endotoxin tolerance is conceptually distinct from immune paralysis and was originally thought of as a protective mechanism against septic shock, its induction might be associated with increased risks of secondary infections.^94^ Circulating monocytes isolated from septic patients show typical characteristics of endotoxin tolerance: loss of HLA‐DR expression, high levels of intracellular inhibitory factors such as interleukin‐1 receptor‐associated kinase M and suppressor of cytokine signaling 1, as well as downregulated tumor necrosis factor alpha production after ex vivo lipopolysaccharide treatment.^95^ Lipopolysaccharide preconditioning attenuates the host inflammatory response in mice but improves bacterial clearance and survival in a polymicrobial model of murine sepsis, possibly by increasing phagocytic activity of macrophages.^96^ Interindividual variations of endotoxin tolerance induction could lead to differential susceptibility to lipopolysaccharide‐triggered inflammatory conditions. Reestablishment of sensitivity to lipopolysaccharide is accomplished by its detoxification from the resolving infection. This process is carried out by a host enzyme, acyloxyacyl hydrolase, which removes fatty acids from lipid A, thereby rendering it inert to toll‐like receptor– or caspase‐based detection.^97^ Interestingly, human periodontal ligament cells and gingival fibroblasts are unable to develop the state of endotoxin tolerance and might play an important role in sustaining the inflammatory response in periodontal disease.^98^ From a clinical point of view, a tailored use of therapeutic interventions that modulate the host inflammatory response and enhance bacterial clearance has obvious potential.

## 6 ENDOTOXEMIA

Translocation of lipopolysaccharide to the bloodstream causes endotoxemia. Metabolic endotoxemia was first defined as high‐fat diet–induced approximately twofold increase in plasma lipopolysaccharide levels due to increased proportions of lipopolysaccharide‐containing microbes in the gut microbiota.^3^ This endotoxemia is associated with low‐grade inflammation.^3^ In metabolic endotoxemia, the levels of lipopolysaccharide are 10‐15 times lower than those seen in sepsis. Similar to those processes seen in local inflammation, lipopolysaccharide triggers the systemic inflammatory response mainly via toll‐like receptor 4. In the circulatory environment, toll‐like receptor 4 is mainly expressed on the surface of immune cells, but also in some nonimmune cells, like endothelial cells.^99^ Binding of lipopolysaccharide to toll‐like receptor 4 leads to the activation of two partially overlapping proinflammatory signaling pathways,^64^, ^100^, ^101^, ^102^, ^103^, ^104^ as presented earlier (Figure 2).

Locally or systemically produced lipopolysaccharide induces inflammatory response to activate the acute‐phase response (Table 1) including C‐reactive protein, leukocyte migration, the complement system, and contact activation pathway aiming at eliminating pathogens.^105^ Different arms of the contact activation pathway lead to increased coagulation, thrombosis, fibrinolysis, hemorrhage, vasoactivity, and inflammation.^106^ Lipopolysaccharide activity also correlates with GlycA,^107^, ^108^ a novel spectroscopic marker of systemic inflammation reflecting both increased glycan complexity and circulating acute‐phase protein levels during local and systemic inflammation.^109^

**TABLE 1:** Association of endotoxemia (serum lipopolysaccharide activity) with demographic factors and cardiometabolic disorders

Group	Parameter	Beta, *P*‐value
	Age, years	**0.037, 0.003**
	Gender	−0.017, 0.173
	Current smoking	−0.011, 0.360
	Number of extracted teeth	**0.031, 0.020**
	Education, years	−0.024, 0.074
	Fasting time, h	−0.001, 0.954
Cardiometabolic disorder	Diabetes mellitus, coronary heart disease, obesity, or metabolic syndrome	**0.178, <0.001**
Obesity	Body mass index, kg/m^2^	**0.213, <0.001**
	Waist/hip ratio	**0.201, <0.001**
	Weight, kg	**0.182, <0.001**
Physical activity	Amount in week, h	**−0.029, 0.021**
	At work	−0.014, 0.264
	Leisure time	**−0.073, <0.001**
Diabetes	Plasma glucose, mg/dl	**0.056, <0.001**
	Prevalent diabetes	**0.058, <0.001**
Hypertension	Prevalent hypertension (American Heart Association definition)	**0.120, <0.001**
	Systolic blood pressure, mm Hg	**0.096, <0.001**
	Diastolic blood pressure, mm Hg	**0.127, <0.001**
	Pulse, n/min	**0.061, <0.001**
Metabolic syndrome	Prevalent metabolic syndrome (International Diabetes Federation definition)	**0.288, <0.001**
	Number of metabolic syndrome components	**0.327, <0.001**
Cardiovascular diseases	Prevalent cardiovascular diseases	**0.030, 0.018**
	Prevalent coronary heart disease	**0.029, 0.019**
	Prevalent acute myocardial infarction	**0.034, 0.007**
Liver function	Carbohydrate‐deficient transferrin, U/L	**0.056, <0.001**
	Alcohol consumption, g/week	**0.038, 0.002**
	gamma‐Glutamyltransferase, U/L	**0.145, <0.001**
Clinical biochemistry	Creatinine, mg/dl	**0.026, 0.040**
	Cystatin C, mg/L	**0.088, <0.001**
	Apolipoprotein‐B, g/L	**0.282, <0.001**
	Apolipoprotein‐A1, g/L	0.016, 0.207
	High‐density lipoprotein cholesterol, mmol/L	**−0.160, <0.001**
	Triglycerides, mmol/L	**0.570, <0.001**
	Cholesterol, mmol/L	**0.285, <0.001**
Inflammation	C‐reactive protein, mg/L	**0.051, <0.001**
	Adiponectin, μg/ml	**−0.086, <0.001**
	Serum amyloid A, mg/L	−0.031, 0.223

Note

All linear models are adjusted for age, gender, and current smoking. *N *= 6782. Originally described in^113^, ^193^ Statistically significant values are in bold.

**FIGURE 4:**
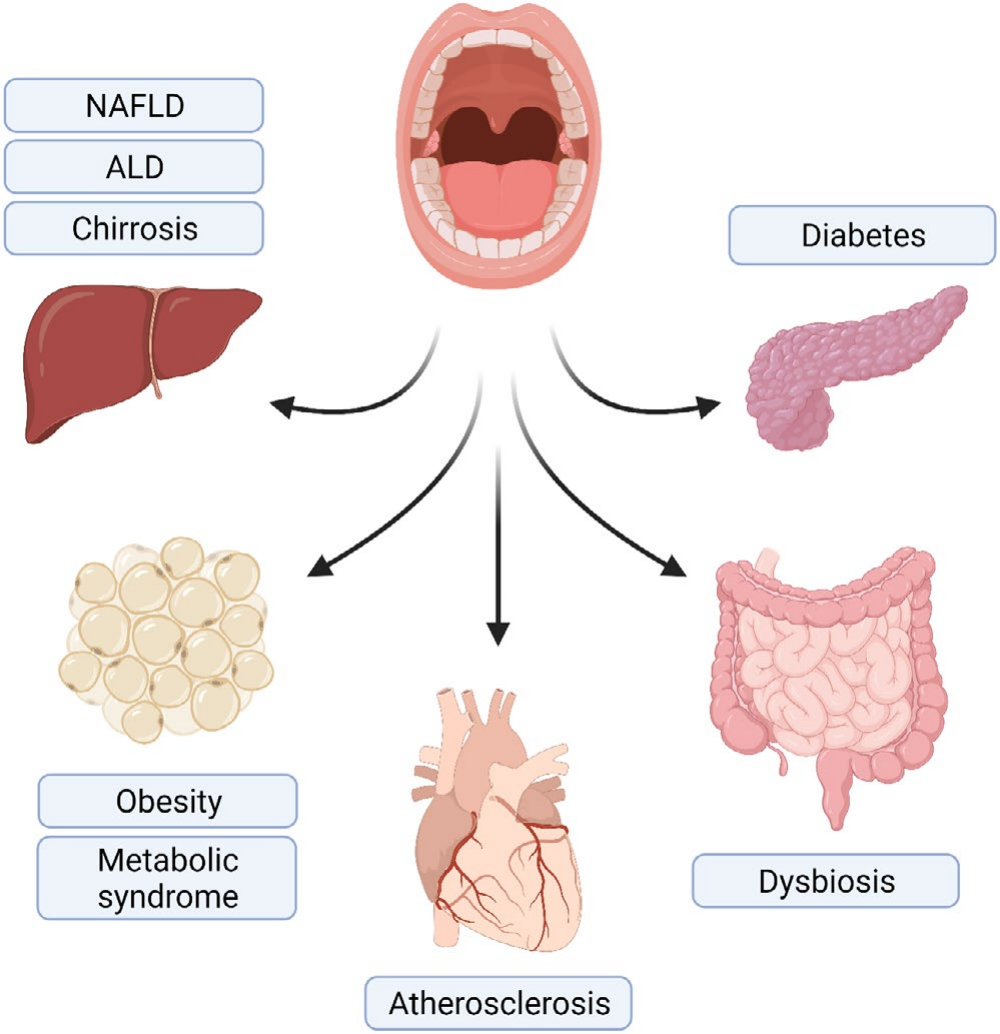
Translocation of lipopolysaccharide from the oral cavity to the circulation leads to endotoxemia, which may affect several organs and contribute to the development of various cardiometabolic disorders. ALD, alcoholic liver disease; NAFLD, nonalcoholic fatty liver disease

Inflammation is an essential component of host defense, but an unresolved chronic low‐grade inflammatory state may lead to a wide range of chronic conditions.^110^, ^111^ Inflammation may derive from endotoxemia, which is a risk factor for chronic cardiometabolic disorders, such as obesity, nonalcoholic fatty liver disease, metabolic syndrome, insulin resistance, type 2 diabetes, dyslipidemia, and cardiovascular diseases^5^, ^8^, ^112^, ^113^, ^114^, ^115^, ^116^ (Figure 4). At least for thromboembolic events and stroke, the association seems to be causal.^117^ Inflammatory markers associated with periodontitis and cardiometabolic disorders largely overlap. Also, molecular mimicry caused by cross‐reactive autoantibodies promotes inflammation linking periodontitis with cardiometabolic disorders.^118^ Transient bacteremia and lipopolysaccharide‐induced endotoxemia have been proposed as potential molecular mediators between periodontitis and cardiometabolic disorders.

In addition to inducing systemic inflammation, bacteria and lipopolysaccharide may have direct effects on the vessel walls and atherosclerotic lesions. Deoxyribonucleic acid of periodontal pathogens (eg, *P. gingivalis*) and live bacteria has been detected in atherosclerotic plaques,^119^, ^120^, ^121^ and experimental studies have shown that lipopolysaccharide is proatherogenic in vitro and in vivo. Potential effects of bacteria and their products include induction of endothelial dysfunction, contribution to the formation of fatty streaks and atherosclerotic plaques, and acceleration of plaque maturation and plaque rupture. Multiple types of inflammatory cells are present in atherosclerotic plaques, and inflammation is a major driver of plaque maturation and rupture.^122^ In atherosclerotic lesions, macrophages and endothelial cells in particular express toll‐like receptor 2 and toll‐like receptor 4 receptors, and toll‐like receptor 4 expression in macrophages is upregulated by oxidized low‐density lipoprotein.^123^, ^124^ Lipopolysaccharide may activate endothelial cells and induce the expression of cytokines and chemokines, tissue factor, and cell adhesion molecules, leading to increased adhesion of leukocytes to the endothelium and increased vascular permeability.^125^, ^126^, ^127^, ^128^ The vascular inflammation activates coagulation and may lead to thrombosis.^129^ *P. gingivalis* lipopolysaccharide facilitates monocyte adhesion to the endothelium in vitro through upregulation of intercellular cell adhesion molecule 1 and vascular cell adhesion molecule 1.^126^ By activation of toll‐like receptors in the inflammatory cells and smooth muscle cells within atherosclerotic plaques, lipopolysaccharide may contribute to atherosclerotic damage via cytokine and prostaglandin induction and oxidative stress.^130^ Lipopolysaccharide may also promote foam cell formation, as macrophages challenged with *A. actinomycetemcomitans* lipopolysaccharide showed enhanced secretion of tumor necrosis factor alpha and IL‐1β and induction of foam cell formation and accumulation of low‐density lipoprotein.^131^ In addition, *A. actinomycetemcomitans* lipopolysaccharide stimulation decreases messenger ribonucleic acid (mRNA) levels of scavenger receptor B, and adenosine‐triphosphate‐binding cassette transporter‐1, which may lead to attenuated cholesterol efflux from foam cells.^131^ Lipopolysaccharide may contribute to the rupture of the fibrous cap of an unstable plaque by stimulating the production of matrix metalloproteinases by, for example, endothelial cells, macrophages, and mast cells within the plaque.^132^, ^133^, ^134^ In a mouse model, injection of lipopolysaccharide accelerated the formation of instable atherosclerotic plaques.^135^

### 6.1 Sources of systemic lipopolysaccharide

Oral pathogens or lipopolysaccharide molecules expressed on their surfaces may enter the lymph and the bloodstream through inflamed gingival tissues. Additionally, some of the species associated with periodontitis, such as *P. gingivalis*, are able to invade and replicate in epithelial cells.^136^ However, the gut microbiota is considered the main source of the lipopolysaccharide in metabolic endotoxemia. The intestinal epithelium is composed of a single layer of intestinal epithelial cells protected by a mucus that forms a physical barrier against pathogenic bacteria. T and B cells, dendritic cells, and macrophages are also present, helping to maintain intestinal homeostasis.^137^ Intestinal epithelial cells are attached to each other, with different junctional complexes forming the intestinal barrier that protects the body against infection and inflammation. Tight junctions are multiprotein complexes composed of actin together with peripheral and integral transmembrane proteins, such as occludins, claudins, and junctional adhesion molecule. The structure of this complex is further strengthened by cytoplasmic scaffolding and adapter proteins.^137^, ^138^

Toll‐like receptor 4 mediates tight junction permeability in the colon.^139^ Interference of intestinal homeostasis by unbeneficial bacterial challenge causes the expression of proinflammatory cytokines, such as tumor necrosis factor alpha and interferon‐gamma, which eventually leads to increased permeability of tight junctions.^140^, ^141^ Lipopolysaccharide contributes further to “leaky gut” by altering the intestinal epithelial tight junction protein assembly, leading to the translocation of lipopolysaccharide from the lumen of the intestine into the bloodstream.^138^

Approximately 1.5 L saliva is swallowed per day linking mouth and gut together. Saliva contains approximately 10^9^ CFU/ml bacteria, and thus also large amounts of lipopolysaccharide with a 10,000‐fold activity compared with serum.^142^ The segregation of oral and intestinal communities was previously thought to be strictly maintained by various mechanisms, such as gastric acidity^143^, ^144^ and antimicrobial bile acids in the duodenum.^145^ Thus, only a failure of this barrier could lead to overgrowth of oral microbes in the gut. However, bacteria detected from oral cavity and stool overlapped in nearly half (45%) of the subjects in the Human Microbiome Project,^45^ and it is estimated that one third of the oral bacteria can survive through the gastrointestinal tract.^146^ Transfer of oral bacteria to the gut is therefore common, but most of bacterial species are not capable of colonizing the colon. However, oral bacteria tolerating the acidic environment in the stomach may proliferate also in the gastrointestinal tract, which seems to be the case with *P. gingivalis* affecting functionality of colon.^147^

### 6.2 Lipoproteins and neutralization

In the circulation, lipopolysaccharide can be recovered in bacterial cell walls, bacterial outer membrane vesicles, bound to bacterial or host proteins, or in blood cells, but the predominant fraction is carried after disaggregation with plasma lipoproteins.^33^ Only 20%‐25% of lipopolysaccharide activity has been reported to exist unbound to lipoproteins.^148^, ^149^ The mean (standard deviation) lipopolysaccharide distribution decreased among lipoprotein classes among periodontal patients as follows: very low‐density lipoproteins/intermediate density lipoproteins 41.3% (12.1%), unbound 25.0% (7.0%), high‐density lipoprotein subfraction 3 13.1% (5.2%), low‐density lipoprotein 11.5% (3.7%), and high‐density lipoprotein subfraction 2 9.2% (2.8%), demonstrating clearly how very low‐density lipoprotein has the lowest and high‐density lipoprotein the highest neutralization capacity.^149^ The triglyceride‐saturated lipoprotein‐lipopolysaccharide complex is eliminated by hepatocytes, preventing lipopolysaccharide‐induced toxicity,^150^ or phagocytosed by macrophages.^151^ The inflammatory state, the lipoprotein profile, and concentrations of lipopolysaccharide‐binding proteins of the subject determinate the metabolic fate of lipopolysaccharide.^8^

Under standard physiological conditions, lipopolysaccharide preferentially associates with high‐density lipoprotein, which contributes to its clearance via the liver and bile.^152^ In the case of low high‐density lipoprotein levels in serum (eg, in septic patients), the majority of lipopolysaccharide is associated with very low‐density lipoproteins.^153^ Similar to during the acute‐phase response,^153^ the majority of lipopolysaccharide activity is recovered in the very low‐density lipoproteins fraction in periodontitis, and the distribution is not substantially affected in a short‐term follow‐up after periodontal treatment.^149^ Despite the clinical success, periodontal treatment did not influence very low‐density lipoprotein composition or the ability to activate macrophages.^150^ However, in a cross‐sectional setting, very low‐density lipoproteins–bound lipopolysaccharide activity and triglyceride content had strong and positive correlations with the cholesterol uptake by macrophages ex vivo. This ability of very low‐density lipoproteins to activate macrophages was higher in patients with stronger signs of inflammation (periodontal pocket depth + bleeding on probing + suppuration, C‐reactive protein, or fibrinogen).^8^ The proportion of lipopolysaccharide bound to very low‐density lipoproteins was positively correlated with the number of deepened periodontal pockets, number of mobile teeth, and C‐reactive protein, whereas lipopolysaccharide bound to either high‐density lipoprotein subfraction 2 or 3 was correlated negatively with these clinical parameters.^154^ Therefore, endotoxemia may depend on inflammatory status, lipoprotein profiles, and concentrations of specific lipopolysaccharide‐transferring proteins.^155^, ^156^

Lipoprotein‐bound lipopolysaccharide is taken up by the liver and excreted in the bile. The lipid transfer/lipopolysaccharide binding protein gene family includes lipopolysaccharide‐binding protein, bactericidal/permeability‐increasing protein, phospholipid transfer protein, and cholesteryl ester transfer protein.^157^ Bactericidal/permeability‐increasing protein inhibits bacterial growth and prevents leukocyte activation by binding to lipopolysaccharide and forming complex directly with the bacterial outer membrane.^158^ Cholesteryl ester transfer protein does not have the ability to transfer lipopolysaccharide, but it has a role in lipoprotein remodeling during inflammation.^159^ Lipopolysaccharide‐binding protein and phospholipid transfer protein are capable of extracting lipopolysaccharide from bacterial outer membrane fragments to high‐density lipoprotein particles,^160^ whereas binding to membrane‐bound cluster of differentiation 14 or soluble cluster of differentiation 14 promotes inflammatory responses. Recently, a lipopolysaccharide neutralizing capacity assay indicated that the individual serum lipopolysaccharide‐neutralizing capacity values may range between 51% and 83% in a population. The main determinants of neutralizing capacity were 1) triglyceride concentration (inverse association), 2) high‐density lipoprotein (direct association) and low‐density lipoprotein cholesterol (inverse association) concentrations and phospholipid transfer protein activity (inverse association).^37^ Interestingly, serum lipopolysaccharide‐binding protein and soluble cluster of differentiation 14 are not correlated with lipopolysaccharide or lipopolysaccharide neutralizing capacity. Serum IgG antibodies to *A. actinomycetemcomitans* predicted high lipopolysaccharide neutralizing capacity with equal responses between different serotypes (from A to E).^37^

### 6.3 Metabolomics

Endotoxemia is tightly connected to lipoprotein metabolism, since lipoproteins are the main carriers of lipopolysaccharide and responsible for neutralization of its biological activity. A major manifestation of aberrant metabolic pathway utilization during inflammatory diseases is the process of lipoprotein remodeling.^161^ Our analyses presented in Table 1 describe the lipid levels in endotoxemia: Endotoxemia has a strong positive correlation with triglyceride, cholesterol, and apolipoprotein B concentrations and a negative correlation with high‐density lipoprotein cholesterol concentration. The association with dyslipidemic lipoprotein phenotype with high serum triglyceride and cholesterol concentrations and low high‐density lipoprotein cholesterol has been observed repeatedly,^5^, ^37^, ^114^ but a broader metabolomics associated with endotoxemia was recently published.^107^ In the largest cohort of the study, endotoxemia presented a significant association with 154/157 (98%) metabolites, demonstrating how widely endotoxemia affects metabolism or vice versa. The first three principal components associating with endotoxemia included (1) very low‐density lipoproteins parameters, apolipoprotein B, and fatty acids, including monounsaturated fatty acids and saturated fatty acids; (2) a large mean diameter of very low‐density lipoproteins, low high‐density and low‐density lipoprotein cholesterol contents, and small high‐density lipoprotein particle size; and (3) high levels of lipid‐rich high‐density lipoprotein particles, fatty acids, monounsaturated fatty acids, saturated fatty acid, and low ratio of apolipoprotein B/apolipoprotein A1. Thus, large very low‐density lipoprotein particle size but small low‐density and high‐density lipoprotein particle sizes promote endotoxemia, indicating how all lipoprotein classes and the subclass particle sizes and compositions are involved. In a meta‐analysis, periodontitis patients had significantly higher low‐density lipoprotein cholesterol and triglyceride and lower high‐density lipoprotein cholesterol concentrations compared with controls, whereas the effect on total cholesterol concentrations had more variation.^162^

Low‐density lipoprotein size or subclass distribution is associated with the severity of periodontitis,^163^, ^164^ showing that small, dense low‐density lipoprotein particles are present among untreated patients. Predomination of this low‐density lipoprotein phenotype, which is associated with endotoxemia,^107^, ^163^ is highly proatherogenic because these particles are especially potent in promoting subendothelial foam cell formation—the hallmark of early atherosclerosis.^122^ Small, dense low‐density lipoproteins have a low affinity to the low‐density lipoprotein receptor, enter the arterial wall readily, undergo oxidative modifications (oxidized low‐density lipoprotein), and enhance cholesterol uptake by macrophages.^165^ The activated macrophages further promote the oxidation of low‐density lipoprotein and its uptake to the cells by releasing reactive oxygen species and oxidative enzymes.^122^ These proatherogenic phenomena were also shown using isolated low‐density lipoprotein preparations from periodontitis patients, where the extent of affected tissue (periodontal pocket depth, suppuration, bleeding on probing) was directly associated with the enhanced cholesterol uptake and production of cytokines by macrophages ex vivo.^166^ The main mediators of these observations were low‐density lipoprotein cholesterol, lipopolysaccharide activity, and modified phospholipids and phospholipid‐binding proteins. These results emphasize the importance of lipoprotein metabolism in the connection between periodontitis and atherosclerosis through lipoprotein remodeling and lipopolysaccharide carriage.

In the metabolomic study, endotoxemia was inversely associated with high‐density lipoprotein particle size, suggesting that large high‐density lipoprotein particles are more efficient in lipopolysaccharide neutralization than small high‐density lipoprotein particles, whose concentration is associated directly with endotoxemia.^107^ High‐density lipoprotein composition also seemed to play a major role in lipopolysaccharide neutralization, since apolipoprotein A1 and the core lipids (ie, triglycerides and cholesteryl esters) were associated with high endotoxemia, whereas the high‐density lipoprotein surface lipids (ie, free cholesterol and phospholipids) were associated with low endotoxemia. These high‐density lipoprotein–related structural determinants are similar to those observed after periodontal treatment, when endotoxemia decreases^166^: an increase in high‐density lipoprotein particle size, and alterations in phospholipid content and subclass distribution; these all lead to a better efflux capacity of the particles from macrophages ex vivo. However, high‐density lipoprotein is also considered anti‐atherogenic due to properties other than its function in reverse cholesterol transport, such as stimulation of endothelial nitric oxide production and anti‐inflammatory, antiapoptotic, and antithrombotic characteristics, which may protect the endothelium directly.^167^ Either due to endotoxemia or independently of it, a combination of decreased concentrations and increased dysfunction of high‐density lipoprotein forms a vicious proatherogenic circle during infection.

Among non–lipid or non–lipoprotein metabolites, the nuclear magnetic resonance metabolomic study^107^ revealed associations between endotoxemia and several measures involved in the risk of cardiometabolic diseases.^168^ These included branched‐chain amino acids, aromatic amino acids, creatinine, and glycolysis, gluconeogenesis, and β‐oxidation‐related metabolites, indicating how widely the lipopolysaccharide‐induced inflammation affects metabolic pathways.

### 6.4 Endotoxemia in periodontitis

Owing to the presence of a large number of gram‐negative bacterial species in subgingival microbiota, patients with periodontitis suffer from endotoxemia and have antibodies against lipopolysaccharide deriving from periodontal pathogens.^163^, ^169^, ^170^, ^171^ The relative amount of 70 different subgingival bacterial species was recently shown to correlate with salivary lipopolysaccharide activity, whereas no association with serum lipopolysaccharide activity was found.^142^ In accordance with the hypothesis, only the quantity of gram‐negative oral species, especially the classical periodontal pathogens, contributed to salivary lipopolysaccharide activity.^142^ Saliva lipopolysaccharide activity had a weak but significant correlation with serum lipopolysaccharide in the whole study population, and this correlation strengthened when periodontally healthy patients were removed from the analysis.^142^ Therefore, saliva lipopolysaccharide can be considered as a merged biomarker of gram‐negative subgingival species. Similarly, saliva lipopolysaccharide is associated with a high cumulative risk score of periodontitis, number of teeth, and alveolar bone loss,^142^, ^172^, ^173^ whereas the association between the cumulative risk score and serum lipopolysaccharide is weaker.^173^ The cumulative risk score is a salivary biomarker for the risk of periodontitis composed of three measurements connected to the periodontal inflammatory process: bacteria, inflammation, and tissue destruction.^174^

Several lipopolysaccharide‐transferring proteins have been detected in periodontitis. Periodontitis is associated with soluble cluster of differentiation 14 levels, which also decreased due to periodontal treatment and predicted the severity of periodontal destruction.^175^, ^176^ Endotoxin levels were higher in patients with localized aggressive periodontitis than in healthy subjects, and the levels correlated with gingival crevicular fluid inflammatory markers and clinical signs of periodontitis.^177^ Serum phospholipid transfer protein activity decreases after periodontal treatment,^166^ whereas its salivary activity does not correlate with any periodontal parameters even though phospholipid transfer protein can be detected in saliva.^178^ Serum lipopolysaccharide‐binding protein concentrations were higher in patients with aggressive periodontitis than in healthy controls.^179^ Lipopolysaccharide‐binding protein concentrations decreased after periodontal treatment in liver cirrhosis patients along with decreasing lipopolysaccharide levels, whereas these levels increased during the 30‐day follow‐up in cirrhosis patients not receiving periodontal therapy.^180^ Importantly, these alterations were accompanied with improved dysbiosis in stool and saliva, underlining the role of local inflammation in the oral‐gut‐hepatic axis.^180^

**FIGURE 5:**
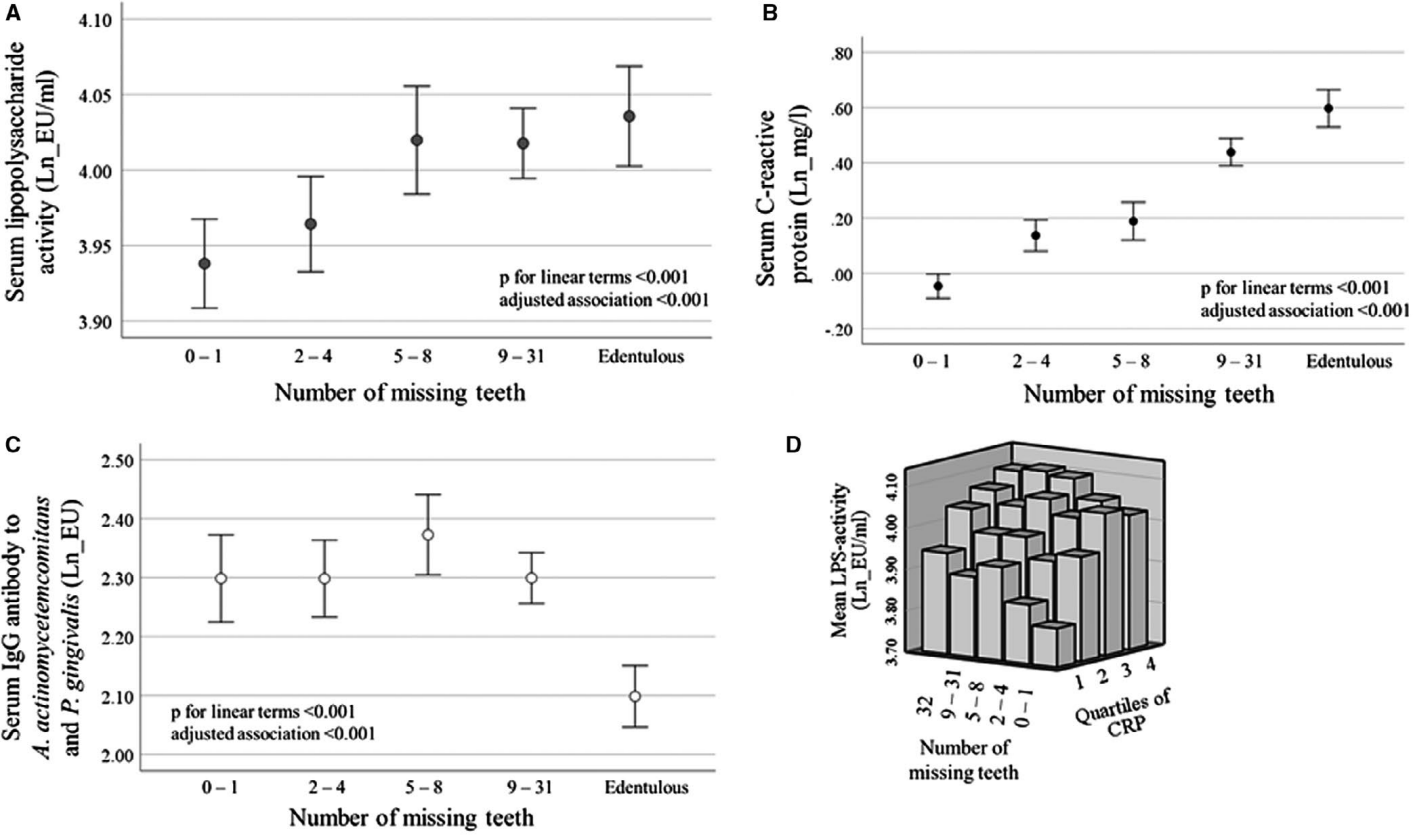
The association of missing teeth with serum lipopolysaccharide activity, C‐reactive protein (CRP), and antibody levels to periodontal bacteria. Serum lipopolysaccharide activity was determined by *Limulus* amebocyte lysate from a population‐based sample of FINRISK‐97 including 6671 participants. A trained nurse counted the number of teeth during the clinical examinations.^228^ Number of participants in the groups divided according to the number of missing teeth are 0‐1 missing teeth, 1440; 2‐4 teeth, 1270; 5‐8 teeth, 883; 9‐31 teeth, 1979; edentulous, 1099. Mean and 95% confidence interval of logarithmically (ln) transformed lipopolysaccharide activity, C‐reactive protein, and immunoglobulin G (IgG)‐class antibodies to *Aggregatibacter actinomycetemcomitans* and *Porphyromonas gingivalis* (combined)^228^, ^229^ are shown. *P*‐values for unweighted linear terms from an ANOVA test and adjusted *P*‐value from a linear regression model adjusted for age, gender, and smoking are presented. EU/ml, endotoxin units; EU, enzyme‐linked immunosorbent assay units

Very low‐density lipoprotein (and low‐density lipoprotein) preparations isolated from periodontitis patients induce macrophages to produce cytokines and to convert into foam cells, connecting periodontitis with atherogenic processes.^154^, ^163^ Endotoxemia may persist despite successful periodontal treatment, and only small alterations can be seen in the lipopolysaccharide distribution among the lipoprotein classes.^149^ Dental extraction causes bacteremia, which is rapidly cleared, and endotoxemia, which is quickly detoxified.^181^, ^182^ However, if the exposure is repeated or continuous, then low‐grade inflammation (Table 1) is sustained, forming a threat to overall health. As seen in Figure 5, the number of missing teeth correlates strongly with both lipopolysaccharide activity and C‐reactive protein. Interestingly, the edentulous subjects have both high lipopolysaccharide activity and high C‐reactive protein, clearly showing that there are also other sources of lipopolysaccharide than the oral cavity. At the same time, edentulous subjects have low antibody levels against periodontal bacteria that derive from dysbiotic oral microbiota^183^ (Figure 5).

## 7 ENDOTOXEMIA AND CARDIOMETABOLIC DISORDERS

### 7.1 Factors associating with endotoxemia

In a population‐based study conducted in Finland (FINRISK‐97), lipopolysaccharide activity was measured by the *Limulus* amebocyte lysate assay from serum samples of 6782 participants.^113^ The study examined possible associations between the measured lipopolysaccharide activity with demographic factors and cardiometabolic disorders as well as related factors. The associations of lipopolysaccharide activity with demographic factors, physical activity measures, liver function, and cardiometabolic disorders are presented in Table 1.

#### 7.1.1 Demographics

Only a modest positive association was observed with age, whereas no association was detected with gender, current smoking, education years, or fasting time before sampling. Physical activity demonstrated a weak negative association with lipopolysaccharide. The association was strongest with the physical activity during the leisure time, although heavy exercise may increase intestinal permeability and endotoxemia.^184^

#### 7.1.2 Genetics

Although endotoxemia has been a hot topic in research for two decades, the role of genetics in the responsiveness to lipopolysaccharide or circulating levels of endotoxemia have been seldom investigated in humans. Common missense mutations in the toll‐like receptor 4 receptor are associated with a phenotype hyporesponsive to inhaled lipopolysaccharide,^185^ and an intergenic cluster located in 7p11.2 is associated with lipopolysaccharide‐induced febrile response.^186^ However, neither of these genetic variations were among those associating with endotoxemia on a genome‐wide level in over 11 000 Finnish subjects.^117^ Altogether, the genome‐wide association study found 741 single‐nucleotide polymorphisms in five independent loci in chromosomes 3, 4, 5, 11, and 15, which explained up to 9.2% of the lipopolysaccharide activity variation. The loci were associated with expression of genes *KNG1* (kininogen‐1), *F11/KLKB1* (FXI/kallikrein), *F12* (FXII), *SERPING1* (plasma protease C1 inhibitor), and *LIPC* (hepatic lipase). Kininogen‐1 is the precursor protein to high‐molecular‐weight kininogen, low‐molecular‐weight kininogen, and bradykinin. Thus, the single‐nucleotide polymorphisms were mainly located at genes that affect the contact activation of the coagulation cascade and lipoprotein metabolism, and, indeed, the composed genetic risk score had a strong association with venous thromboembolism. Activation of the kallikrein‐kininogen pathway enhances the production of the vasodilator bradykinin. It has been hypothesized that bradykinin release in the intestinal tract could decrease gut barrier function and promote translocation of endotoxins from the intestinal lumen to the circulation.^187^

#### 7.1.3 Nutrition

A high‐fat diet has been associated with increased intestinal permeability and metabolic endotoxemia.^188^ Lipopolysaccharide is able to cross the gastrointestinal mucosa, and it has a high affinity to chylomicrons.^189^ Therefore, lipopolysaccharide has been suggested to be the molecular link between a high‐fat diet, the microbiota, and inflammation.^3^ According to several interventions among healthy individuals, high‐fat and/or high‐carbohydrate and/or energy‐rich meals lead to endotoxemia.^8^ The postprandial increase of circulating endotoxin may be stronger in subjects with metabolic disorders, such as impaired glucose intolerance or type 2 diabetes,^190^ but conflicting results have also been published. When patients with type 1 diabetes and nondiabetic controls were given high‐caloric, fat‐containing meals for 1 day, they had only a modest effect on serum lipopolysaccharide activity, although profound changes in chylomicron and high‐density lipoprotein metabolism as well as serum cytokine levels were observed.^191^, ^192^ In a large Finnish study including a dietary 24‐hour recall, lipopolysaccharide was significantly associated with cardiometabolic disorders (obesity, metabolic syndrome, diabetes, coronary heart disease events) independently of established risk factors, C‐reactive protein, and intake of total energy, macronutrients, protein, fat, or fibre.^193^ However, in multivariate models, lipopolysaccharide activity was associated directly with total energy intake and indirectly with available carbohydrates only in lean, healthy subjects, distinguishing the responses between healthy and metabolically compromised participants.

Dietary patterns are crucial in shaping the gut microbiota, and they contribute to gut permeability and thereby endotoxemia.^194^ A high‐fat diet enriched with both saturated and unsaturated fatty acids has an impact on the gut barrier by several direct and indirect mechanisms,^194^ and high‐carbohydrate diets lead to loss of microbial diversity and sucrose‐induced dysbiosis in the gut,^195^ both promoting endotoxemia. Saturated fatty acids, especially lauric acid (C12:1), are toll‐like receptor 4 agonists,^196^ leading to a similar inflammatory response as lipopolysaccharide. In turn, experimental studies suggest that *n*–3 polyunsaturated fatty acids inhibit the activation of toll‐like receptor 4 by disrupting the signaling or translocation of toll‐like receptor 4 into lipid rafts, thereby displaying anti‐inflammatory effects.^197^, ^198^, ^199^ An unhealthy diet is a known risk factor for caries and periodontal disease,^200^ but the oral microbiota seems to be more resilient to dietary effects compared with that of the large intestine.^201^

### 7.2 Outcomes

On the one hand, the outcome of chronic endotoxemia and continuous low‐grade inflammation may be cardiometabolic disorders, which are commonly connected to each other. On the other hand, these disorders promote intestinal permeability and systemic inflammation, accelerating the process of deteriorating systemic health. Virtually all cell types are irritated by the endotoxin challenge, which thereby affects tissue types widely (**Figure** **4**).

#### 7.2.1 Liver diseases

The healthy liver expresses low levels of toll‐like receptor 4, whereas lipopolysaccharide and toll‐like receptor 4 signaling have been proposed to play a role in the pathogenesis of alcoholic liver disease, nonalcoholic fatty liver disease, and nonalcoholic steatohepatitis.^202^ In a prospective population‐based cohort, high serum lipopolysaccharide activity predicted incident advanced liver disease.^203^ As expected in FINRISK‐97 (Table 1), lipopolysaccharide was associated with gamma‐glutamyltransferase, an important diagnostic marker of liver disease and a predictor of incident diabetes,^204^ whereas the association was weak with measures of alcohol consumption by a questionnaire and carbohydrate‐deficient transferrin. Although lipopolysaccharide may induce liver diseases, its source has been seldom linked to the oral cavity. *P. gingivalis* has been detected more frequently in the oral samples of nonalcoholic fatty liver disease patients than in control subjects.^205^ In mouse studies, gram‐negative bacteria, such as *A. actinomycetemcomitans*, infected the liver and caused proatherogenic alterations and dyslipidemia.^206^ *A. actinomycetemcomitans* was detected in the liver accompanied by infiltration of neutrophils, and increased triglycerides/phospholipids ratio and inflammatory gene expression. The pathogen administration also led to induction of *A. actinomycetemcomitans* antibodies, serum amyloid A, and lipopolysaccharide in the circulation.^206^ *P. gingivalis* has also been shown to aggravate nonalcoholic steatohepatitis in a mouse model by free fatty acid–induced NLR family pyrin domain containing 3 inflammasome activation and lipopolysaccharide‐toll‐like receptor pathway.^205^, ^207^ In vitro, *P. gingivalis* lipopolysaccharide induces lipid accumulation and inflammation in hepatic cells.^208^

#### 7.2.2 Obesity and metabolic syndrome

A high‐fat/high‐energy diet was shown to induce modest elevations of endotoxemia (1.5‐fold) in lean mice, and this elevation was accompanied with increased fat deposition, systemic inflammation, and insulin resistance.^3^ The adipose tissue was considered as the source of low‐grade inflammation frequently observed in obese individuals; but after the previously mentioned mouse studies, the increased gut permeability has been implicated in systemic inflammation. Adipose tissue, however, is an active, multifunctional metabolic organ composed of heterogeneous cell populations. Dysfunction of the adipose tissue modifies immune responses by free fatty acids and adipokines and contributes to metabolic disorders.^209^ Recurrent infection of mice by *A. actinomycetemcomitans* has been shown to result in marked changes in the fatty acid composition of both inguinal and epididymal adipose tissue; these alterations had a strong correlation with serum lipopolysaccharide activity.^210^

Metabolic syndrome is defined based on the presence of five criteria, which include an elevated waist circumference, triglyceride level, fasting glucose blood concentration, and blood pressure and reduced high‐density lipoprotein cholesterol concentration. Endotoxemia is associated with obesity and with separate components and the presence of metabolic syndrome.^3^, ^113^, ^114^ This is also seen in the results presented in Table 1, where lipopolysaccharide activity associated strongly with body mass index, waist/hip ratio, weight, number of metabolic syndrome components, and prevalent metabolic syndrome (Table 1). During disturbed metabolism, such as in metabolic syndrome, the unbeneficial associations between endotoxemia, lipid metabolism, and inflammation may be even stronger than in metabolically healthy subjects.^107^

#### 7.2.3 Diabetes

Subjects with diabetes are more susceptible to fungal and bacterial infections compared with the general population. Many patients suffer from persistent skin, urinary tract, and oral infections at some point during their lifetime. It is also a well‐recognized fact that elevated glucose levels in blood increase the risk of acute and chronic infections. High blood glucose variability, which is usually associated with poor glycemic control, correlates with the use of antibiotics in individuals with diabetes. Based on earlier studies, each 1% increase in hemoglobin A1c has been associated with 3%‐10% higher rate of antibiotic purchases.^211^, ^212^ Since diabetes is commonly associated with dysregulation of the immune system, the patients are more susceptible to infections caused by opportunist pathogens; for example, *Staphylococcus aureus* (skin infections), *E. coli* (urinary infections), *Pseudomonas aeruginosa* (pneumonia), and *P. gingivalis* (periodontitis). Compared with nondiabetic subjects, individuals with type 1 or type 2 diabetes have generally two to four times higher risk for hospitalization due to bacterial infections.^213^ Fecal biomarker and microbiome analyses have revealed that patients with diabetes have decreased levels of beneficial microbes (eg, anaerobic butyrate‐producing bacteria).^214^, ^215^, ^216^, ^217^

Long diabetes duration also increases the risk of micro (nephropathy, retinopathy, neuropathy) and macrovascular (cardiovascular diseases, including stroke) complications.^218^ Based on the Finnish registry data on the use of antibiotics, bacterial infections seem to be much more common in diabetic subjects with existing renal, cardiovascular, and retinal complications.^116^, ^211^, ^219^ It should be noted that many of these subjects with severe infections might already have a long history of chronic inflammation—reflected by increased levels of circulating proinflammatory compounds (eg, cytokines, C‐reactive protein).^220^ High serum endotoxin activity could be one of the explanations for the persistent low‐grade inflammation, especially in subjects with existing diabetes‐related complications.^114^, ^116^, ^212^, ^219^ Metabolic endotoxemia is one of the risk factors for diabetes and its associated complications.^113^, ^193^, ^221^, ^222^ In the FINRISK population presented in Table 1, lipopolysaccharide activity was associated directly with plasma glucose concentration and prevalent diabetes. It was also associated with creatinine and cystatin, markers of impaired renal function.

#### 7.2.4 Cardiovascular diseases

Endotoxemia is associated with cardiovascular diseases, such as myocardial infarction (MI), incident coronary artery disease events, and stroke.^5^, ^112^, ^115^, ^193^ The hazards for incident cardiovascular disease events were greater when a subject with high endotoxemia had at the same time low high‐density lipoprotein cholesterol, or high C‐reactive protein or interleukin‐6.^5^ An association with prevalent cardiovascular diseases was also seen among the analyses presented in **Table** **1**.

In several studies, circulating concentrations of lipopolysaccharide‐transferring proteins have also been used as a proxy of endotoxemia. Compared with healthy controls, higher lipopolysaccharide‐binding protein and soluble cluster of differentiation 14 concentrations have been observed in patients with ischemic stroke,^115^ and the concentrations were especially high in patients with a poor short‐term prognosis.^223^ In another study, however, lipopolysaccharide‐binding protein or soluble cluster of differentiation 14 were not correlated with lipopolysaccharide activity or serum lipopolysaccharide‐neutralizing capacity in patients with ischemic stroke or their matched controls.^37^ Genetic polymorphisms contributing to serum lipopolysaccharide activity were associated with the risk of “ischemic stroke,” “any stroke,” “TOAST small artery occlusion,” “TOAST cardioaortic embolism,” and “intracranial aneurysm” in the Megastroke population, whereas the genetic risk score of lipopolysaccharide activity presented associations with deep vein thrombosis, pulmonary embolism, and venous thromboembolism.^117^ Even a causal association of lipopolysaccharide in stroke was suggested in this study, which is the first one exploring the genetic background of endotoxemia.^117^

### 7.3 Animal studies

The influence of *P. gingivalis* on the gut microbiome and colon functions has been further studied in mouse models. Orally administered *P. gingivalis* caused increased levels of plasma endotoxin and insulin and reduced mRNA expression of the tight junction protein zonula occludens‐1 in the small intestine of C57BL/6N mice. In addition, microbiome analysis showed that the amount of Bacteroidales was significantly increased compared with sham‐treated mice.^224^ As *P. gingivalis* was not among the bacterial species detected in the blood of the *P. gingivalis* administered mice, it was speculated that *P. gingivalis* contributed to the development of endotoxemia by affecting the composition of gut microbiota, leading to increased intestine permeability.

The same phenomenon was also seen in another study with C57BL/6N mice, where even a single administration of *P. gingivalis* caused disturbance in the gut microbiota with an increased level of Bacteroidetes and decrease of Firmicutes.^225^ The mRNA expression of the tight junction proteins involved in maintaining intestinal barrier was downregulated. No outgrowth of *P. gingivalis* was detected in the gut, and the amount of Porpyromonadaceae in the fecal samples was less than 0.003%, suggesting further the indirect effect of *P. gingivalis* on the development of gut‐derived endotoxemia.

## 8 DISCUSSION AND CONCLUSIONS

Undoubtedly, lipopolysaccharide is a potent and multifaceted activator of inflammation and immunological responses. Additionally, it is clear that inflammation is associated with diverse metabolic alterations designed to neutralize the invading microorganisms, minimize the extent of tissue damage, contribute to tissue regeneration, and replace proteins involved in the inflammatory process. For example, increases of triglyceride‐rich lipoproteins during inflammation provide lipid substrates for the activated immune system, whereas decreases of high‐density lipoprotein help to conserve cholesterol at peripheral sites where areas of injury may need extra cholesterol for new membrane synthesis. However, these alterations are harmful in long‐term chronic inflammatory conditions, such as periodontitis.

Lipopolysaccharide may enter the bloodstream through inflamed periodontal tissues, especially after dental treatment,^163^, ^181^ and even after gentle mastication or tooth brushing.^170^ Moreover, saliva contains huge amounts of lipopolysaccharide, which retains its biological activity following protease treatment or low pH, thereby contributing to toll‐like receptor stimulants of the small intestine.^226^ However, the evidence that periodontitis‐associated dysbiosis contributes to endotoxemia is not as strong as in the case of gut microbiome dysbiosis. Regardless of the source of systemic lipopolysaccharide, lipopolysaccharide‐associated systemic inflammation may additionally cause long‐term metabolic and epigenetic rewiring in hematopoietic stem cells, leading to sustained enhancement of inflammatory myelopoiesis that may aggravate both cardiometabolic disorders and periodontitis.^227^ Although metabolic endotoxemia is regarded to result from translocation of lipopolysaccharide from the gut, the gut and the mouth are not separated. They are physically, microbiologically, and biochemically connected to each other, and only crumbs of information are known about this connection. Although the causality is insufficiently demonstrated thus far, many studies have associated changes of the gut microbiome composition, function, and specific bacterial metabolites with cardiometabolic diseases,^194^ whereas research on the oral microbiome has severely fallen behind. However, evidence is accumulating that dysbiosis is a disease affecting the whole body, from head to toe.
